# Geographical and Ecological Drivers of Zoonotic Viral Spillover: A Review of Emerging and Re-emerging Outbreaks

**DOI:** 10.7759/cureus.99820

**Published:** 2025-12-22

**Authors:** Mahendra Verma, Harjeet S Maan, Shravya Konatam, Yogendra Verma, Rohit Kumar, Deepti Chaurasia, Lokendra Dave, Shweta Sharma

**Affiliations:** 1 Research and Development, Centre of Excellence (CoE) National Institute of Pharmaceutical Education and Research (NIPER) Raebareli, Lucknow, IND; 2 Department of Microbiology, State Virology Laboratory, Gandhi Medical College, Bhopal, IND; 3 College of Arts and Sciences, Nova Southeastern University, Davie, USA; 4 Department of Life Sciences, Mandsaur University, Mandsaur, IND; 5 Department of Microbiology, Avalon University School of Medicine, Willemstad, NLD; 6 Department of Respiratory Medicine, Gandhi Medical College and Hamidia Hospital, Bhopal, IND; 7 Department of Pediatrics, Gandhi Medical College, Bhopal, IND

**Keywords:** cross-species transmission, ecological boundaries, emerging infectious diseases, one health approach, zoonotic viral spillover

## Abstract

Over the past two decades, outbreaks of zoonotic viruses have become increasingly frequent and severe, posing substantial threats to public health systems and the global economy. The viruses responsible for these outbreaks, such as SARS-CoV, MERS-CoV, Zika, Ebola, Nipah, avian influenza, and, most recently, SARS-CoV-2, typically originate in wildlife, highlighting the complex relationship between ecological systems and human activities. Human-wildlife interactions have markedly increased due to disruptions in environmental and geographic boundaries, primarily driven by urbanization, deforestation, intensified agricultural practices, and climate change. These factors contribute to an environment that facilitates zoonotic transmission spillover.

This narrative review summarizes current research on the ecological, geographic, and human factors influencing zoonotic virus transmissions. It emphasizes how these viruses adapt to human hosts and cross species barriers via direct contact, vector-borne transmission, intermediate carriers, and environmental contamination. Moreover, the review discusses how the genomic plasticity of viruses enhances their transmissibility and facilitates adaptation to new hosts, thereby increasing the risk of epidemics and pandemics.

The review further underscores the importance of ecological boundaries in mitigating spillover events and advocates for a One Health approach that integrates human, animal, and environmental health. This approach is essential for predicting, detecting, and preventing future outbreaks. In conclusion, the review emphasizes the importance of interdisciplinary research, proactive surveillance, habitat preservation, and policy interventions that address the underlying ecological factors contributing to zoonotic outbreaks. Restoring ecological barriers and implementing sustainable practices to minimize the interaction between wildlife and humans, while bolstering global biosecurity, are essential measures to mitigate the risk of future pandemics.

## Introduction and background

The twenty-first century has witnessed several zoonotic disease outbreaks, which are infectious illnesses transmitted from animals to humans. Many of these outbreaks have had significant global impacts, highlighting the complex connections between human and animal populations [1]. Research from 1970 onward has clearly shown that, out of approximately 1,500 recorded diseases, nearly 70% originated from animals (wildlife) [2]. Furthermore, the World Health Organization identified 15 viruses of zoonotic origin that pose a worldwide threat to human health. Among these pathogens, respiratory viruses are the most common, with the potential for causing both epidemic and endemic outbreaks [3]. Moreover, zoonotic pathogens are not limited to causing respiratory infections; they also cause other deadly diseases, such as HIV and the Zika virus. Over the past twenty years, zoonotic viruses, including SARS-CoV, MERS-CoV, Ebola, Influenza, and COVID-19, have affected the global population, leading to significant global mortality. COVID-19 alone has resulted in over 7 million fatalities worldwide [4]. The primary zoonotic pathogens impacting the global population include SARS-CoV, MERS-CoV, Zika, Ebola, Nipah [5], and Influenza, along with the more recent SARS-CoV-2 and Monkeypox [6], as discussed here (Table 1). It is important to remember that most zoonotic viruses originate from a common ancestor: wild animals. Given the recent and sharp rise in zoonotic viral infections in humans, understanding how pathogens spread from wildlife to humans across different regions is crucial [7]. Over the past few decades, human-wildlife interactions have advanced beyond points where ecological and geographic barriers once contained these viruses, now permitting them to cross species boundaries [8,9]. Does restoring barriers between wildlife and domestic animals help prevent future viral outbreaks?

**Table 1:** Major zoonotic viral outbreaks in the twenty-first century.

S. no.	Virus/Disease	Year of emergence	Region(s) affected	Primary host/Reservoir	Estimated death toll	References
1	Influenza A (H1N1)	2009	Global	Swine (Pigs)	~284,000 deaths (est.)	[1]
2	Ebola Virus	2014-2016, 2018-2020	West Africa, DRC	Fruit Bats, Non-human Primates	~11,000 deaths	[1,2]
3	Zika Virus	2015-2016	Americas, Pacific Islands	Mosquitoes (Aedes spp.), Primates	~3,500 microcephaly cases	[3]
4	SARS-CoV-2 (COVID-19)	2019-Present	Global	Likely Bats, Unknown Intermediate Host	>7 million deaths	[4,9]
5	Nipah Virus	1998-Present	Malaysia, Bangladesh, India	Fruit Bats (Pteropus spp.)	~700+ deaths	[5]
6	Monkeypox Virus (Mpox)	2022 (Global re-emergence)	Africa, Europe, Americas	Rodents, Primates	~100+ deaths (2022-2023)	[6]
7	MERS-CoV	2012	Middle East, South Korea	Bats, Camels	~858 deaths	[9]
8	SARS-CoV	2002-2003	China, Global	Bats, Civet Cats	~774 deaths	[10,11]

## Review

Zoonotic virus

Over the last two decades, the world has witnessed outbreaks of several viruses with the potential to become endemic, epidemic, or pandemic, with most of these viruses originating from zoonotic sources [10]. Zoonotic viruses are viruses that can be transmitted between animals and humans. These viruses could cross species barriers, causing infections in both animals and humans. Zoonotic transmissions can occur through direct contact with infected animals, the consumption of contaminated food or water, or exposure to vectors such as mosquitoes or ticks that carry the virus between species [11]. While some zoonotic viruses may only cause minor infections, others can lead to severe conditions that have a significant impact on public health. It is essential to investigate the variables and causes that contribute to the transmission of viruses from wild types to humans, as evidenced by the re-emergence and emergence of zoonotic viral infections and their ability to infect humans despite ecological limits [12]. It is noteworthy to mention that the viral genome is highly unstable, and changes influence genomic plasticity in the environment. Zoonotic viruses pose challenges for public health and require a coordinated One Health approach, involving collaboration between human and animal health sectors, as well as environmental scientists [13]. Monitoring, surveillance, and research are crucial for understanding the dynamics of zoonotic diseases and preventing their spread. The ongoing COVID-19 pandemic, caused by the SARS-CoV-2 virus, is another example of a zoonotic virus that likely originated in bats with an unknown intermediate host [14].

Animals, especially birds and mammals, naturally carry many viruses. Typically, these viruses don't cause symptoms or only minor ones in their animal hosts, as they have coevolved with them [15]. Many viruses found in wild animals have the potential to infect humans. These viruses are pretty diverse. A study by Pearce-Duvet highlights that bats harbor a wide range of viruses and have been linked to several zoonotic outbreaks [16]. Wild animals act as natural reservoirs for various infections, making them a key part of the ecology of these viruses. Typically, zoonotic viruses that transfer from animals to humans originate in wildlife. To prevent and control the spread of infectious diseases, understanding the relationships between animals and these viruses is essential [11]. Human interaction with natural environments, often beyond ecological limits, is a significant factor driving the emergence, reappearance, and re-emergence of zoonotic viruses as they cross species boundaries [17]. These zoonotic events occur when viruses that naturally infect animals, mostly wildlife, spill over to humans. Such events can lead to the emergence of new human diseases and occasionally result in large outbreaks [18]. To effectively prevent and mitigate the impact of zoonotic events, it is essential to understand these factors and closely monitor high-risk areas and species. Addressing the complex dynamics of viral spread and zoonotic transmission requires a One Health approach, which recognizes the interconnectedness of human, animal, and environmental health [19]. Public health efforts, research, and surveillance are vital tools to control and stop the spread of zoonotic illnesses. Several factors contribute to the occurrence of zoonotic transmission and subsequent viral outbreaks.

Crossing species boundaries and zoonotic transmission

According to Zumla and Hui, close contact with wildlife constitutes a critical determinant in the occurrence of viral spillover events. Regardless of whether the interaction is direct or indirect, it significantly elevates the risk of zoonotic viral transmission [20]. Human encroachment into natural habitats through activities such as hunting, farming, and other anthropogenic actions further increases human proximity to wildlife [21]. The presence of wildlife itself also heightens the risk of zoonotic spillover, as numerous studies have demonstrated that wildlife functions as a natural reservoir for zoonotic viruses and other pathogenic microorganisms [22]. It is noteworthy that these zoonotic viruses are typically nonpathogenic to the wildlife hosts; however, alterations in host species can pose severe health threats, potentially leading to the emergence of life-threatening diseases [23]. For instance, bats are recognized as a reservoir for zoonotic viruses with pandemic potential. The transmission of zoonotic viruses largely depends on intermediate hosts, which can carry the viruses with epidemic and pandemic potential [24]. Notably, intermediate hosts can amplify the virus, thereby facilitating its transmission to humans. An example of this is the 2002-2003 SARS-CoV outbreak, where civet cats were identified as intermediate hosts. The consequences of globalization and urbanization, which result in the loss of wildlife habitats, further exacerbate the risk of zoonotic virus spillover [25].

Urbanization and globalization have gradually led wildlife to venture outside their usual habitats, which can sometimes result in zoonotic viral spillovers. Another challenge in our modern era is food scarcity, which has prompted a rise in the popularity and expansion of animal husbandry over recent decades [26]. It has been reported that intensive farming practices, especially when animals are kept in close quarters, can create conditions conducive to the spread of zoonotic infections from animals to humans, for example, swine flu and avian flu. Additionally, changes to natural ecosystems, including deforestation and climate change, impact the distribution and behavior of wildlife, often increasing interactions with domestic animals [27]. Genetic modifications can also enhance viruses' ability to infect new hosts, including humans, as they evolve.

This evolution is driven by factors such as genetic variations, selection pressures, and the fitness landscape, which collectively heighten the risks of pandemics, drug resistance, and expanding host ranges. As viruses and their variants evolve, the likelihood of zoonotic transmission increases-consider examples such as SARS-CoV-2, avian influenza, and Ebola, where viral evolution has demonstrated a broad host range [28]. The use of antimicrobial drugs in agriculture can also lead to the development of drug-resistant viral strains, posing additional challenges.

Zoonotic transmission pathways****

Robert and Baylis examined how human activities, such as deforestation, urbanization, and agricultural development, constitute significant factors in habitat loss, which in turn facilitate viral spillover and frequent outbreaks [11]. Habitat destruction is a critical element that not only precipitates viral spillovers but also enables species jumps and transmission to humans. In a study, Zumla and Hui demonstrated that disease outbreaks within wildlife populations can often originate from other animal species [20]. To mitigate the occurrence of recurrent viral spills, it is essential to preserve wildlife ecosystems. It is reported that within these ecosystems, particular species serve as reservoirs for viruses that possess pandemic potential, such as bats. Coronaviruses are naturally found in bat habitats, and previous reports have indicated that outbreaks of SARS-CoV and SARS-CoV-2, as well as filoviruses such as the Ebola virus, are associated with these reservoirs. Notably, despite the presence of viruses in bats, their immune systems confer protection against disease. According to a study by Spencer et al., viral spillover is a widespread phenomenon whereby viruses transmit from one host species to another, including humans [19]. In cases of viral spillover, the route of transmission may vary, with direct interaction between hosts and humans being particularly significant. Other mechanisms of transmission include fomites, aerosols, and water. Although viral spillover has posed a considerable threat to human health over the past two decades, there has been a notable increase in instances where viruses have crossed species barriers to infect humans, resulting in various diseases [9]. Understanding the ecology of wildlife hosts and the factors influencing zoonotic transmission is vital for early detection of outbreaks and the implementation of appropriate measures [29].

The ongoing research focuses on the dissemination and dynamics of viruses, which affect communities. Although wildlife naturally hosts viruses with pandemic potential, transmission to humans requires either an intermediate host or occurs through aerosolized particles and contaminated surfaces, known as fomites. The transmission of zoonotic viruses with pandemic potential depends on the mode of transmission. Kampf et al. demonstrated that intermediate hosts or vectors facilitate not only the rapid propagation of viruses but also subsequent infections [30]. Fomite and aerosol transmission represent supplementary pathways for viral dissemination, particularly hazardous within enclosed and indoor environments. Although numerous environmental samples may test positive for the presence of the virus, such findings do not definitively indicate active infections. Consequently, comprehensive efforts have been made to elucidate the mechanisms of viral spread from various sources, encompassing both clinical and environmental contexts [31]. Molecular surveillance, encompassing genomic and epidemiological analyses, is currently being conducted worldwide to clarify viral dynamics and the connections within transmission pathways from sources to humans. Transmission from clinical sources requires a high viral load of active viruses to initiate infection. The SARS-CoV outbreak of 2002 was traced to a bat origin; however, its primary dissemination occurred through an intermediate host, specifically civet cats. Vector-borne transmission, which can be introduced into medical settings by infected patients, can significantly contribute to disease spread in regions where these vectors are present throughout the year. A study by Rahman et al. demonstrated the critical roles played by vectors such as mosquitoes, fleas, ticks, as well as rats and other rodents in propagating numerous diseases, including viral infections and illnesses [32].

When inanimate objects contaminated by an infected person come into contact with an animal or person who is susceptible to the disease, this is known as fomite transmission. The surfaces in COVID-19 were identified as a high-risk zone for viral dissemination. Exam tables, cages, kennels, medical equipment, environmental surfaces, clothing, and other items may all be categorized as fomites [33]. The term *aerosol transmission *pertains to the dissemination of illnesses via minute particles or droplet nuclei. Aerosol particles may settle on mucosal membranes or environmental surfaces, or they may be inhaled by a susceptible host [34]. Such transmission can occur during specialized medical procedures, including suctioning, bronchoscopy, dentistry, and inhalation anesthesia, or when an infected individual breathes, coughs, sneezes, or vocalizes. Air currents within a room or facility can disperse small particles, which can remain suspended in the environment for prolonged durations [35]. Nonetheless, most pathogens relevant to veterinary care in companion animals are diminutive and are not capable of surviving for extended periods in the environment; consequently, they are unlikely to spread disease through close or prolonged contact.

Ecological boundaries and virus spillover

**Figure 1:**
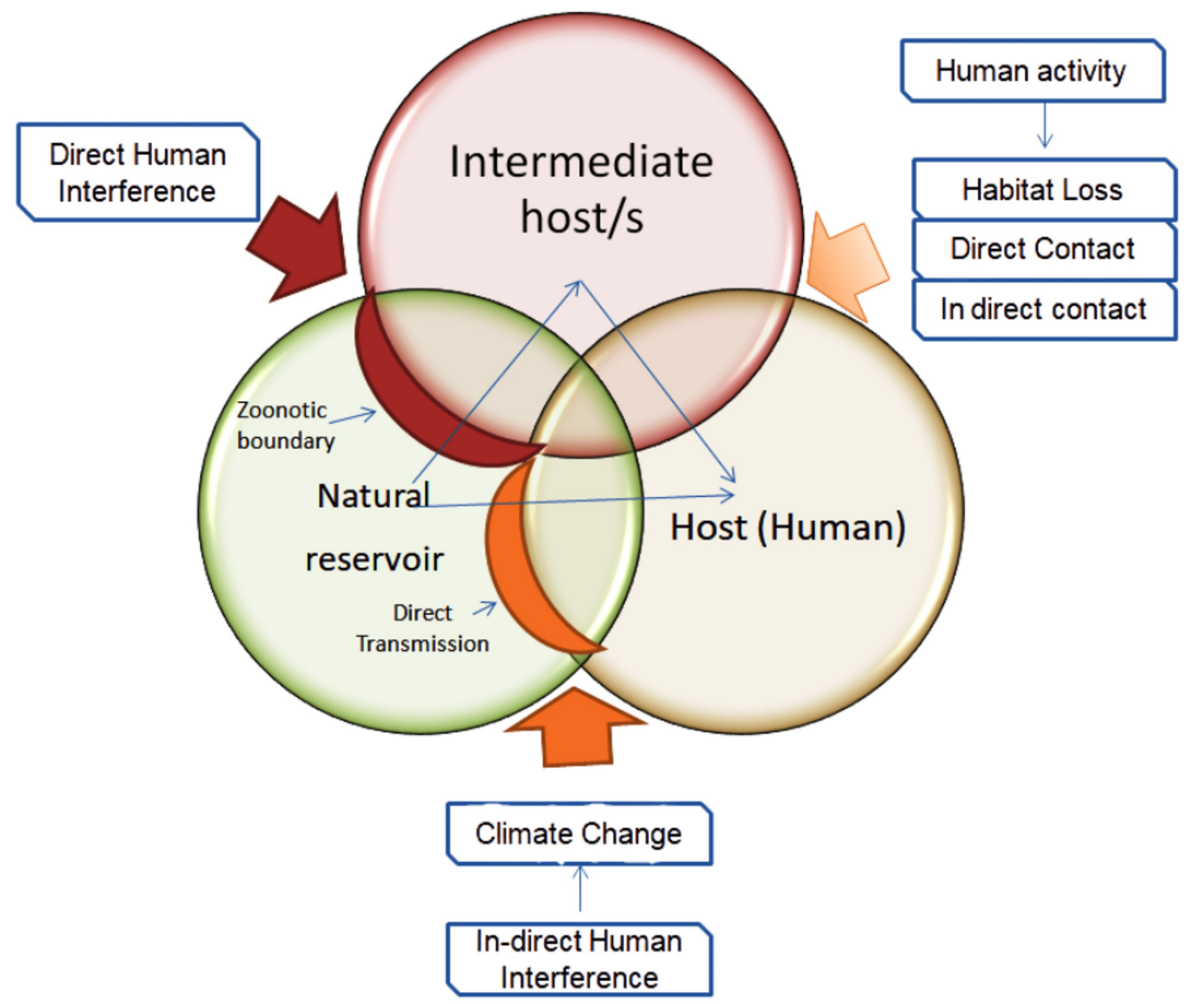
An overview of the interconnections between human activities and zoonotic spillover, highlighting the various mechanisms involved in the zoonotic process. An Illustration of the zoonotic spillover process and mechanism, and engagement of various drivers. Further, the figure decipher the role of geographical and ecological boundaries in the emergence and reemergence of zoonotic infections to the human. In the zoonotic process, the spillover of the pathogen from the reservoir to the host has been illustrated. The figure also enumerates the drivers, such as human engagements and climate changes in progressing zoonotic spillover. Image created by the authors, Dr. Mahendra Kumar Verma and Dr. Harjeet Singh Maan, using Microsoft Artwork tools.

Ecological boundaries and changes within ecosystems can significantly influence the dynamics of viral outbreaks, particularly in the context of zoonotic diseases transmitted from animals to humans [11]. Various ecological factors contribute to the emergence and dissemination of infectious diseases, and a comprehensive understanding of these dynamics is crucial for effective prevention and mitigation of outbreaks. A recent study by Holmes elucidates the ecology of viral emergence and the role of ecological boundaries in the rapid appearance and reemergence of viral pathogens [12]. When an animal's migration results in species crossing, it is often straightforward to identify cases involving clear pathology. Zoonosis refers to the transmission of viruses from non-human hosts to humans [36]. Unpredictable events, involving complex interactions between the virus and the newly adopted host, occur during species crossings (Figure 1). Notable examples of viruses that have crossed the species barrier and become established within the human population include HIV and the contemporary human influenza virus, which do not necessarily require the initial animal reservoir to persist [37]. Fortunately, it is uncommon for a new virus to adapt naturally and spread extensively among humans. Frequently, the virus encounters difficulties in successfully infecting and transmitting between individuals. Human activities that alter the environment, technology, and ecological systems often precipitate the emergence of novel viral infections [38].

These activities may lead to an increase in human-animal contact with animal hosts that act as reservoirs for zoonotic viruses [39]. Agricultural development, increased exploitation of environmental resources, population growth and mobility, and the trade and transportation of food and livestock have all been linked to the emergence and spread of several new viruses throughout the human population [40]. It seems plausible that the COVID-19 pandemic has led to increased awareness of the growth in zoonotic illnesses. Therefore, this awareness may also skew our knowledge of the ecology and evolution of viruses, particularly concerning the direction and timing of host-jumping events [41]. Understanding the processes that lead to disease onset and averting future zoonotic catastrophes requires acknowledging that viruses have been an integral part of global ecosystems for a long time, long before they became clinically or agriculturally significant [42]. This understanding also underscores the close relationship between ecological disturbance and the onset of disease. As humans become a larger part of the global ecosystem that contains viruses, the key issue is not merely that zoonotic viruses infect humans; rather, it is the increasing frequency and how modern human society influences it. When viruses are viewed through the lens of ecosystems, the widely accepted idea of One Health expands [43]. The ecosystems perspective is more expansive than One Health, which focuses primarily on viruses as disease agents, particularly those that connect illnesses in humans and animals [28].

Considering all major viruses, including those that have adapted to coexist harmoniously with their hosts, as well as the variables that disrupt ecosystems and increase the likelihood of disease outbreaks, along with the complex repercussions that follow ecological disturbances [24]. An essential principle of the ecosystem approach is that, although zoonotic virus transmission to humans is a common and regular occurrence, disease outbreaks are infrequently caused by this process. While the primary causes remain unclear, metagenomic research consistently demonstrates that healthy animal species can harbor a diverse array of viruses without exhibiting apparent health effects [44]. Traditionally, virologists have predominantly focused on studying viruses that infect humans, domesticated animals, or plants of significant importance to human society. Although this anthropocentric perspective is understandable, it may have contributed to a limited understanding of the scope and interconnectedness of the global virosphere [45]. The term *zoonosis *inherently implies directionality: viruses possess animal reservoirs, representing the source populations, and then transmit to humans as novel hosts. This view naturally considers humans as the endpoint of an evolutionary process. Although all respiratory viruses infecting humans ultimately originate from those found in other animals, humans are not the sole recipients of these viruses [46]. 

The endemic barrier associated with cross-species migration, which relates to the emergence of infectious diseases, has been extensively examined [47]. From micro and macro perspectives, these barriers represent two aspects of the ecological barrier that influence the probability of emerging viruses spreading within human populations. The cross-species barrier signifies the infrequency with which viruses effectively transmit among novel hosts that have not previously been exposed to them or are not susceptible [44]. Viral mutation or evolution is principally responsible for breaching the cross-species barrier, facilitating spillover infections into other hosts. Such events enable viruses to gradually adapt to new host cells and ultimately disseminate into additional populations. Conversely, overcoming the endemic barrier depends on the likelihood and frequency of viral transmission, which is closely linked to interactions between natural hosts of viruses and humans or potential hosts [28]. Despite numerous investigations into early outbreaks and epidemics of infectious diseases and their association with cross-species or endemic barriers [39], a comprehensive and systematic analysis of viral migration and transmission within ecosystems from a macro perspective remains lacking. These investigations mainly emphasize epidemiology and immunology.

The precise zoonotic origin of viruses, such as those with pandemic potential (coronavirus and influenza), possesses a higher degree of genomic plasticity that facilitates molecular evolution. During the COVID-19 pandemic, rapid surges of several variants were observed in a short period. Several factors, including host, environment, and therapeutics, affect viral genomic plasticity and its molecular evolution. Additionally, there are natural mechanisms by which microbes regularly change their genome for improved survival. He et al. note that genomic plasticity is primarily influenced by a changing environment, where human interference plays a critical role [38]. Human interference with wildlife causes habitat loss, where constant climate change shows the emergence and reemergence of viral outbreaks. Therefore, by altering interactions between humans and the natural environment, increased human activity may reduce ecological barriers and accelerate the spread of viruses within human civilizations [48]. Zhang et al. demonstrated how human interference breached the ecological barrier that serves as a protective boundary between wildlife and humans, thereby increasing the possibility of zoonotic viral spillover [36]. More precisely, the four main ecological barrier elements essential for viral transmission, which act as a molecular or endemic barrier, are transmission pathways, contact likelihood, contact frequency, and viral characteristics. The environmental barrier integrates all possible obstacles to viral transmission from the virus's natural or intermediate hosts to human society, making it a vital hub for recently developing infectious illnesses [37].

One Health approach to tackle viral spillover

The relationships among animal, human, and environmental health are particularly evident in the context of zoonoses [38]. These refer to illnesses or infections that vertebrate animals can naturally transmit to humans. The emergence or re-emergence of pathogens can occur through various mechanisms. For example, the *migration* of known pathogens to new regions (e.g., the Ebola virus spreading to West Africa) and the adaptation of pathogens to new hosts (e.g., the H5N1 avian influenza virus adapting to humans) exemplify these mechanisms [25]. At the microscopic level, emergence and re-emergence involve genetic modifications that may lead to increased resistance to antimicrobial agents. Therefore, drivers of emergence can impact our environment at multiple scales, potentially resulting in pathogenic alterations that give rise to novel disease entities [28]. A critical challenge in recognizing and detecting newly emerging zoonoses is inherently linked to this broad scope. Additionally, biological factors are not the sole contributors. The initial human case marks the point at which a new disease from an animal reservoir host transmits to humans. This juncture is likely to influence the subsequent dissemination of disease within human populations, considering factors such as ecology, sociology, and human and animal behaviors [49]. The One Health approach is an interdisciplinary framework that acknowledges the interconnectedness of human, animal, and environmental health in addressing complex health issues, such as the prevention and control of viral spill-over events. It emphasizes collaboration across various sectors, including public health, veterinary medicine, environmental science, and human medicine [50]. 

In circumstances where surveillance and early detection systems for both human and animal health, in conjunction with environmental surveillance, require an integrated approach, a One Health strategy may serve as a pivotal preventive instrument to mitigate viral spillover. It is also imperative to undertake collaborative studies with specialists from various disciplines to understand the ecology of viruses, their natural reservoirs, and the factors influencing spillover events. To enhance collective knowledge and early warning mechanisms, fostering the exchange of data and information across environmental, animal, and human health sectors is revolutionary [25]. Additional measures are essential, such as identifying high-risk regions with sensitive ecological interfaces and assessing areas or ecosystems with significant potential for viral spillovers, considering variables including wildlife diversity, human-wildlife interactions, and land-use changes. The implementation of risk-reduction strategies, such as sustainable land management, wildlife management, and habitat conservation, is vital. Cross-species diagnostics constitute another health strategy that addresses ongoing viral spillover events [51]. To facilitate early detection of potential spillover incidents, it is necessary to develop and adopt diagnostic techniques capable of identifying and detecting viruses in both humans and animals. This approach helps pinpoint high-risk populations and supports surveillance efforts in communities where the likelihood of viral spillover is heightened, particularly those residing in regions undergoing ecological changes or close to wildlife habitats [52].

## Conclusions

Wildlife serves as a natural reservoir of viruses and remains confined within an ecological niche. Zoonosis is a phenomenon that permits viruses to transfer from native species to humans, often via an intermediate host. Zoonosis does not occur spontaneously; instead, it is driven by various factors and drivers that promote viral spillover, with human interference in wildlife ecosystems being a key factor. In wildlife populations, viruses generally do not cause significant harm to their reservoir hosts. The evolution of wildlife and viruses is a mutually beneficial process that often occurs without detrimental effects on the host (reservoir). Over the past two decades, since the beginning of the twenty-first century, the world has witnessed successive emergences and re-emergences of viral outbreaks with the potential to cause epidemics and pandemics. Examples such as SARS-CoV-2 and COVID-19 exemplify some of the most severe cases of viral spillover in human history. Natural viral hosts include predators (which transmit viruses through biting or ingestion of intermediate hosts) and parasites (such as ticks or fleas), which facilitate the spread of viruses to other wild animals, known as intermediate hosts. During adaptation and evolution, viruses can infect and spread within these new intermediate hosts, thereby extending natural virus reservoirs into broader environmental domains. This process effectively breaches ecological barriers and endangers human societies by increasing the number of wild animals and parasites capable of transmitting emerging infectious diseases. To establish a more effective system, it is essential to consider risk factors at the interface between humans, animals, and the environment, as well as the sources of zoonotic disease outbreaks. Moreover, legal coherence between environmental treaties and animal health regulations must be enhanced. By reducing the likelihood of pathogen spillover, a One Health approach-promoting and safeguarding the health of animals and the environment-also enhances biosecurity in food production, ultimately benefiting both human and animal health.
